# Pyroptotic cell death in the R6/2 mouse model of Huntington’s disease: new insight on the inflammasome

**DOI:** 10.1038/s41420-020-00293-z

**Published:** 2020-07-31

**Authors:** Emanuela Paldino, Vincenza D’Angelo, Giuseppe Sancesario, Francesca R. Fusco

**Affiliations:** 1 IRCSS Fondazione Santa Lucia, Laboratory of Neuroanatomy, Via del Fosso di Fiorano, 64, 00143 Rome, Italy; 2 Department of Systems Medicine, Tor Vergata University of Rome, via Montpellier 1, 00133 Rome, Italy

**Keywords:** Cellular neuroscience, Huntington's disease

## Abstract

Mechanisms of tissue damage in Huntington’s disease involve excitotoxicity, mitochondrial damage, and neuroinflammation, including microglia activation. In the present study, we investigate the role of pyroptosis process in the striatal neurons of the R6/2 mouse model of Huntington’s disease. Transgenic mice were sacrificed at 4 and 13 weeks of age. After sacrifice, histological and immunohistochemical studies were performed. We found that NLRP3 and Caspase-1 were intensely expressed in 13-week-old R6/2 mice. Moreover, NLRP3 expression levels were higher in striatal spiny projection neurons and in parvalbumin interneurons, which are prone to degenerate in HD.

## Introduction

Pyroptosis is a Caspase-1 (Casp-1) dependent programmed cell death that leads to a rapid lysis of the cell^1^. Similar to apoptosis, pyroptosis is a programmed cell death but is dependent on a different caspase. Pyroptosis indeed shares several features with apoptosis, but at a closer look the two processes are found to be fairly distinct. In pyroptosis, DNA damage occurs and TUNEL assay is positive^2,3^. However, while the apoptotic nucleus becomes pyknotic and subsequently breaks up in karyorrhexis, the nucleus in pyroptosis undergoes a chromatin condensation, but remains intact and karyorrhexis does not occur^4^.

Inflammasomes are complexes of proteins that are assembled in response to various cellular stresses, and their activation leads in turn to the recruitment and activation of procaspase 1^2^. The major components of the inflammasome complex are pattern recognition receptors (PRRs)^5^. Several inflammasomes have been described, which include NLR family pyrin domain containing 3 (NLRP3), NLRP1, AIM2, and others. Among them, NLRP3 inflammasome was described to display a major role in inflammatory and immune system‐related disorders^6^, and also in the pathogenesis of several neurodegenerative diseases^7,8^.

Thus, the interest in this topic is based on its association with various diseases which is leading to the development of therapeutics that target inflammasome activity^9^. With this in mind, the aim of our work was to investigate the relevance of pyroptosis in the neuropathology of Huntington’s disease. In fact, such devastating neurodegenerative disease is still lacking a valid therapy, and we speculate that pyroptosis could represent a new target for future therapies. Thus, in this study, we carried out a research to investigate the presence of pyroptosis in the striatal cells of R6/2 Huntington’s disease mouse model by comparing it to the distribution of apoptosis.

## Results

In order to evaluate the development and the (activation of the different inflammasome components that suggest the involvement of pyroptosis) presence of inflammasome and the involvement of pyroptosis in the neuropathology of HD, we chose to study two different time points, i.e. the early presymptomatic stage of 4 weeks and the fully symptomatic 13 weeks.

### Caspase-8 and NLRP3 expression in the R6/2 mice striatum

**Fig. 1:**
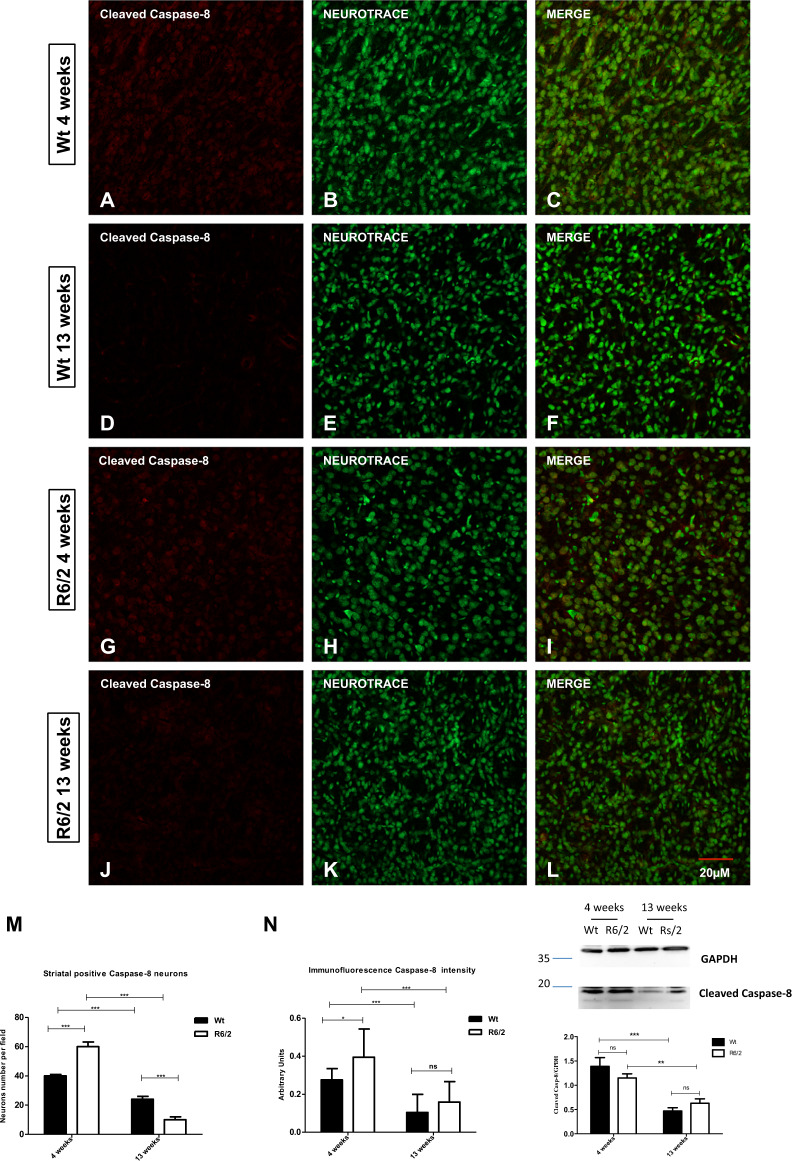
Caspase-8 distribution in striatal neurons. Confocal laser scanning microscopy images of double-label immunofluorescence for CASP-8 (visualized in red fluorescence) and the Nissl-like fluorescent marker Neurotrace (visualized in green fluorescence). **a**–**l** Images show the expression levels of the canonical apoptotic marker Caspase 8 in the striatum of 4 and 13 weeks old wild-type mice and in 4 and 13 weeks old R6/2 mice. **m**, **n** Bonferroni analysis revealed a significant decrease of striatal cleavage product of Casp-8 positive neurons number, as evaluated by western blotting in 13-week-old Wt and R6/2 mice [genotype effect *F*1,36 = 6554; *P* < 0.05; Time effect *F*1,36 = 35,89; *P* < 0.001].

**Fig. 2:**
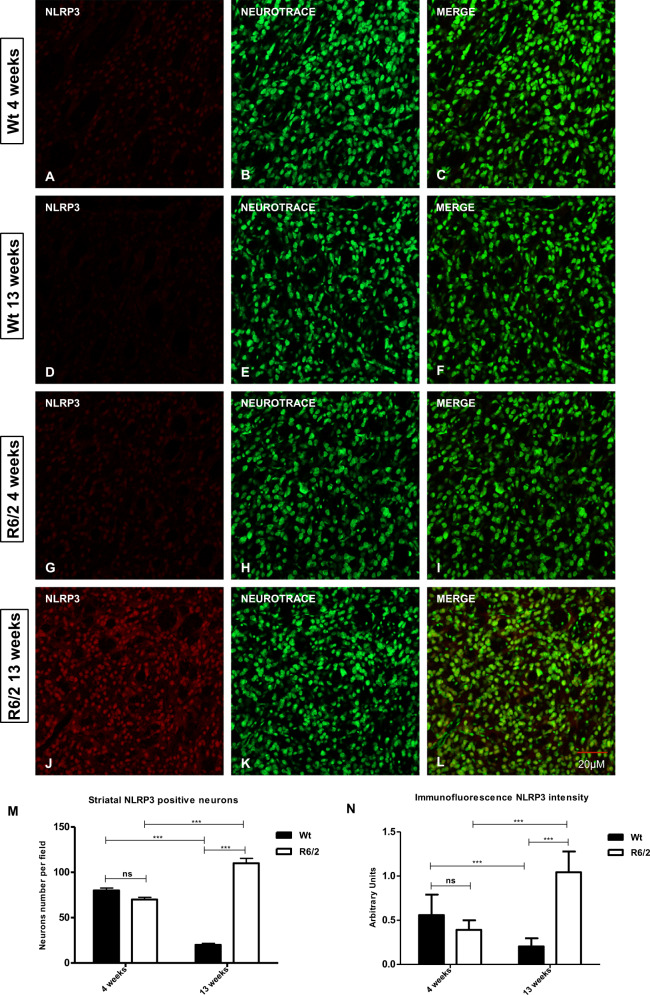
NLRP3 immunostaining in striatal neurons. NLRP3 is showed in red fluorescence, Nissl-like fluorescent marker Neurotrace visualized in green. **a**–**l** Immunoreaction intensity of NLRP3 in each animal group at the different time points. Two-way ANOVA analysis performed on data obtained by 4 and 13 weeks old Wt and 4 and 13 weeks old R6/2 mice showed a statistically significant effect of Genotype (*F*1,36 = 108,9; *P* < 0.001), Time (*F*1,36 = 20.00; *P* < 0.001) and Genotype X Time Interaction (*F*1,36 = 268,9; *P* < 0.001) of NLRP3 positive striatal neurons. **m**, **n** Histogram shows NLRP3 intensity quantification, that is significantly increased in the striatal neurons of 13 week-old R6/2 mice [Genotype effect *F*1,36 = 34,96; *P* < 0.001; Time effect *F*1,36 = 6,830; *P* < 0.05; Genotype X Time Interection *F*1,36 = 78,04; *P* < 0.001].

We studied the distribution of the activated apoptosis marker Caspase-8 (Fig. 1) compared to the inflammasome marker NLRP3 (Fig. 2) in the mice striatum. The number of striatal neurons positive for Caspase-8 appeared higher in the young R6/2 mice than in 13 weeks old R6/2 mice *P* < 0.001 (Fig. 1m), as well as a significant expression of Caspase-8 was also present in 4-week-old Wt (Fig. 1a–c). Cleaved Caspase-8 product at the different time points was also confirmed by Western Blotting analysis. These data suggested that Caspase-8 activation at this stage was linked to the cell homeostasis. In fact, the expression of cleaved Caspase-8 was significantly reduced in the 13-week-old Wt and R6/2 mice *P* < 0.001(Fig. 1n). Immunofluorescence analysis revealed that 13-week-old R6/2 mice had a significantly higher expression of NLRP3 in the striatal neurons compared to the apoptosis marker Caspase-8 (Fig. 2); *P* < 0.001.

### NLRP3 contributes to Caspase-1 activation in the R6/2 mice striatum

**Fig. 3:**
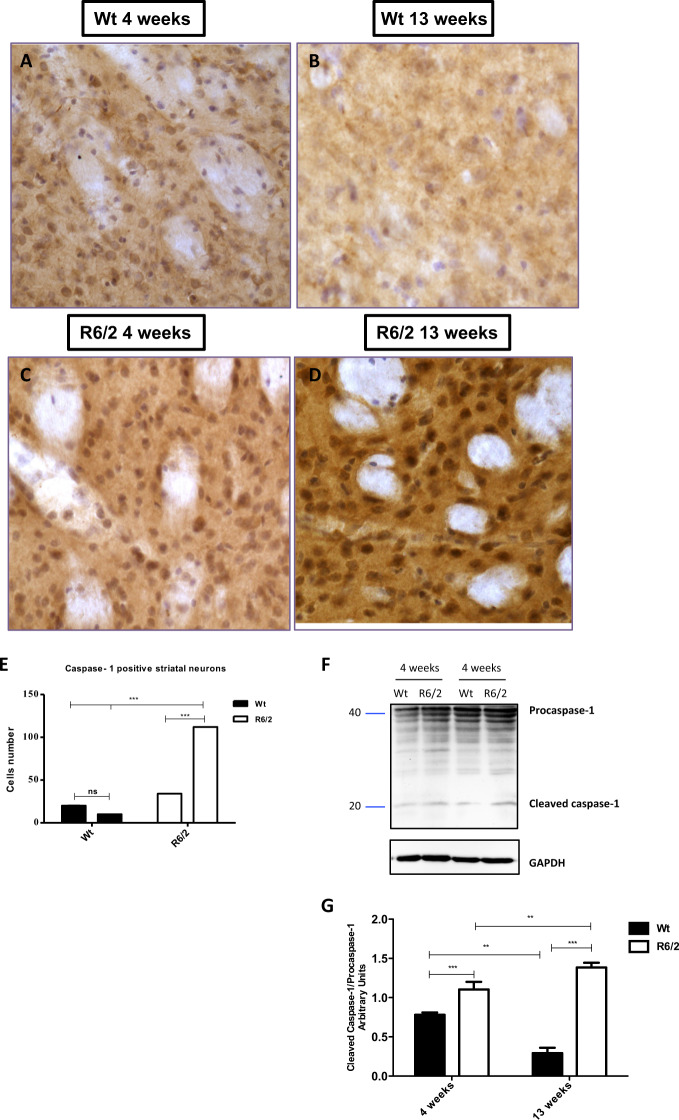
Caspase-1 activation in striatal neurons of Wt and R6/2 mice. **a**–**d** Representative transmitted light microscope images showing Dab staining for Caspase-1 contourstained with Hematoxylin in the striatum of 4- and 13-week-old Wt mice and 4- and 13-week-old R6/2 mice. **e** Two-way ANOVA revealed a statistically significant effect of genotype (*F*1,36 = 18030; *P* < 0.001); Time (*F*1,36 = 6196; *P* < 0.001) and genotypeXtime interaction *F*1,36 = 10,380; *P* < 0.001 in the 13-week-old R6/2 mice compared to 4 weeks old R6/2 mice and littermates. **f**, **g** Representative image of Western Blot and densitometric analysis of cleavage product of Caspase-1. *Bonferroni* analysis performed on probed membranes revealed a significant increase of the striatal cleavage product of Caspase-1 in the 13-week-old R6/2 mice [genotype effect *F*1,20 = 635,9; *P* < 0.001; Time effect *F*1,20 = 13,85; *P* < 0.01] compared to 4- and 13-week-old Wt.

Caspase-1 immunohistochemistry was performed for the detection of pyroptosis (Bergsbacken et al.^10^) in striatal neurons of all mice from each experimental groups. Immunostaining for Caspase-1 in 13-week-old R6/2 mice revealed an intense immunohistochemical reaction, where neurons positive for Caspase-1 were significantly more numerous compared to their wild-type littermates *F*1,36 = 18,030; *P* < 0.001(Fig. 3a–e).

Protein analysis, performed by western blot, revealed the expression of procaspase-1 in all the experimental groups, however, in the R6/2 at 13 weeks of age the cleavage product of procaspase-1 was significantly higher compared to the other groups (Fig. 3f).

### NLRP3 immunoreactivity in Calbindin positive neurons

**Fig. 4:**
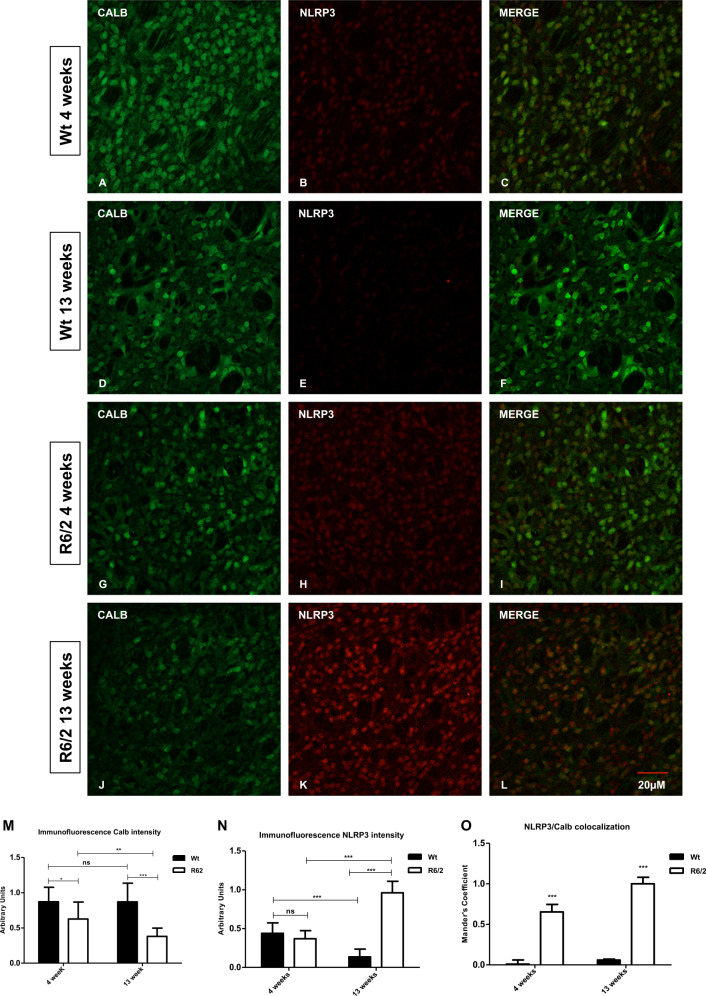
NLRP3 distribution in striatal projection neurons. Confocal microscopy-acquired images of double-label immunofluorescence for Calbindin and NLRP3. Calbindin is showed in green fluorescence, NLRP3 is labelled in red. **a**–**l** Images show the immunoreaction intensity of Calbindin and NLRP3 in each experimental group. Calbindin positive neurons and intensity were dramatically decreased in projection neurons of 13 weeks old R6/2 mice *F*1,36 = 27,57; *p* < 0.001. **m**, **n** Two way ANOVA analysis performed on data obtained by 4 and 13 weeks old Wt and 4 and 13 weeks old R6/2 mice showed a statistically significant effect of Genotype (*F*1,36 = 108,9; *P* < 0.001), Time (*F*1,36 = 20.00; *P* < 0.001) and GenotypeXTime Interection (*F*1,36 = 268,9; *P* < 0.001) on NLRP3 positive striatal neurons. **o** Quantitative analysis of coexpression levels by Mander’s coefficient in all experimental groups. Histogram represented the mean of ROIs calculated Mander’s coefficients revealed the statistically significant colocalization of NLRP3 in Calbindin positive neurons *P* < 0.001.

We investigated the distribution of the NLRP3 in the striatal projection neurons, labeled by Calbindin immunoreactivity. Calbindin labeled striatal projection cells were present in the 13- week-old Wt mice where they constituted the majority of neurons, whereas they were dramatically reduced in the R6/2 mice. Very low levels of NLRP3 immunoreactivity were present in 4-week-old Wt, and no immunoreactivity at all was observed at 13 weeks (Fig. 4a–f). Conversely, the double immunostaining revealed an early suffering of calbindin positive striatal projection neurons in the 4-week-old R6/2 mice as is showed in the graph (Fig. 4m), where we observed a statistically significant decrease in number and in immunofluorescence intensity in 13-week-old R6/2 mice compared to 4- and 13-week-old Wt (Fig. 4j–l). Such specific decrease of calbindin labeled projection neurons and immunofluorescence intensity was associated with the overexpression of NLRP3 in the 13-week-old R6/2 as shown in Fig. 4n. Coloc 2 analysis revealed a statistically significant colocalization of NLRP3 in calbindin neurons in the R6/2 mice compared with both 4- and 13-week-old Wt mice (Fig. 4o).

### Analysis of NLRP3 expression levels in the interneurons subtypes

**Fig. 5:**
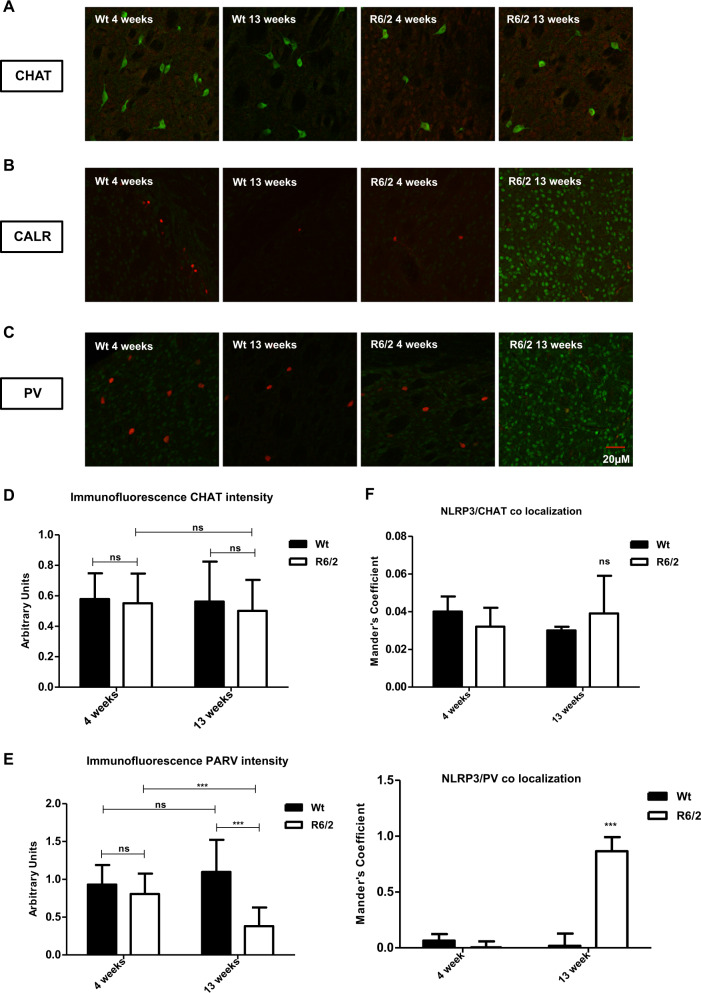
NLRP3 immunostaining in striatal interneurons. Distribution analysis of microglia (Iba-1) and NLRP3 in the striatum mice. Representative confocal images showing the distribution of microglia in the four experimental animal groups (**a**–**d**). **e** Statistical analysis (*t*-test) showed that Iba-1 positive cells increased significantly in the 4 and 13 weeks old R6/2 mice compared to Wt at 4 and 13 weeks of age (*F*(1, 36) = 88, 17; *P* < 0.001), correlating with the statistically significant expression levels of NLRP3. **f** Significant increase of microglial cells area in the 4 and 13 weeks old R6/2 mice compared to Wt at the 2 different time point (genotype effect *F*(1, 36) = 5590; *P* < 0.001; time effect *F*(1, 36) = 132.5; *P* < 0.001).

Along with the distribution of NLRP3 in projection neurons, we studied the pattern of its localization in the different interneurons subtypes, each of which express a peculiar susceptibility to HD degeneration. The intensity of NLRP3 immunoreaction product in the interneurons subtypes was investigated in all experimental groups (Fig. 5a–c). We confirmed a significant reduction of CALR interneurons in the 13-week-old R6/2 mice, where NLRP3 expression, in terms of intensity of immunoreactivity, was significantly increased (Fig. 5b). In the cholinergic interneurons, which are resistant to the specific neurodegenerative process in HD^11,12^, we observed no colocalization of NLRP3 (Fig. 5a, f). Our immunolabeling study showed, in fact, an immunofluorescence intensity of Chat interneurons that was comparable between R6/2 and WT at both time points (Fig. 5d). No NLRP3 immunoreactivity was observed in those interneurons (Fig. 5f). Our study revealed an initial expression of NLRP3 in neurons containing parvalbumin in the 4-week-old R6/2 mice. NLRP3 expression levels culminated in the 13-week-old R6/2 mice in which, also, the number and the immunofluorescence intensity of parvalbumin containing neurons resulted statistically significant decreased (Fig. 5e). The mean calculated Mander’s colocalization coefficient was near 1 (Fig. 5f). In the 4- and 13-week-old Wt mice, in which there was no statistically significant expression of NLRP3, parvalbumin neurons did not decrease in number.

### Microglia activation in R6/2 mice

**Fig. 6:**
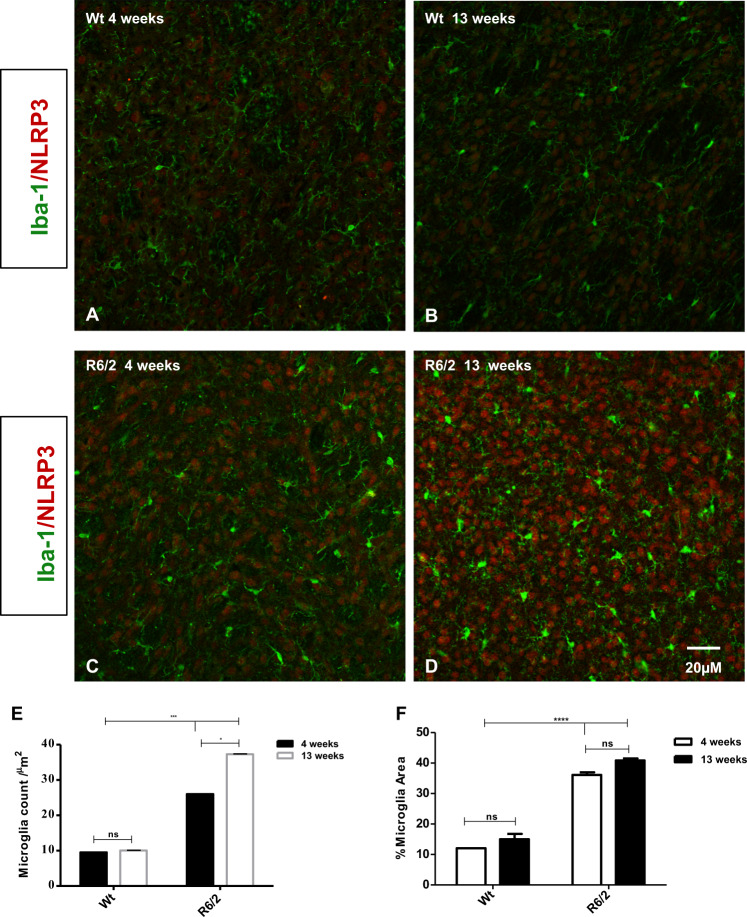
Distribution analysis of microglia (Iba-1) and NLRP3 in the striatum mice. Representative confocal images showing the distribution of microglia in the four experimental animal groups (**a**–**d**). **e** Statistical analysis (*T*-test) showed that Iba-1 positive cells increased significantly in the 4- and 13 week-old R6/2 mice compared to Wt at 4- and 13 weeks of age *F*(1,36) = 88,17 *P* < 0.001, correlating with the statistically significant expression levels of NLRP3. **f** Significant increase of microglial cells area in the 4- and 13 week-old R6/2 mice compared to Wt at the 2 different time point [Genotype effect *F*(1,36) = 5590; *P* < 0.001; Time effect *F*(1,36) = 132.5; *P* < 0.001].

Iba-1 immunofluorescence was performed to study the activation of microglia at different time points. Both at 4 and at 13 weeks, wild-type mice displayed a ramified or primed microglia morphology, with a bigger cell body, but with processes similar to that of ramified microglia. The presence of dystrophic microglia in the 13-week-old-wild-type mice, on the other hand, can be attributed to the aging/senescence process. The immunostaining for Iba-1 in 4 and 13 weeks R6/2 group revealed an intense microglial reaction, where microglial cells appeared numerous and displayed an ameboid cell body in which are still present few ramified or unramified processes (Fig. 6b, c). Microglial reaction increased over time, concurrently with the significantly higher expression levels of NLRP3 in other cells of the striatum. However, unlike previously described^13^, we did not observe NLRP3 immunoreaction in the Iba-1 labeled microglia^14^.

## Discussion

Anomalous apoptosis has been reported to play a role in Huntington’s disease pathology for many years^15^^–17^. Apoptotic cells and DNA degradation products were observed both in HD patients brains^15,16,18^ and in experimental HD models^16,19,20^. Moreover, our group previously described the neuroprotective effects exherted by Poly (ADP-ribose) polymerase 1 (PARP-1) inhibition in the mouse R6/2 model^21^, that are postulated to be mediated by an inhibition of apoptosis^22^. An emerging role for non-apoptotic programmed cell death has been described in neurodegenerative diseases in most recent years^23^. Pyroptosis is an inflammatory form of regulated necrosis which is alternative to apoptosis^23^. Recent studies have shown that pathways of cell death are associated in different ways according to the activation of signaling cascades inducing apoptosis, necroptosis, and pyroptosis^24^. An increasing body of evidence suggests that receptor interacting protein kinase 1 and caspase-8 are the mediators of the cross-talk between apoptosis, necroptosis, and pyroptosis and determine the type of cell death induced in response to activation of cell death signaling^25^.

In this paper, we investigated the presence and behavior of pyroptosis in the R6/2 mouse model of Huntington’s disease^26^.

The comparative study of the distribution of selected pyroptosis and apoptosis markers in the striatum showed that young mice, both wild type and R6/2, express high levels of the cleavage product of Caspase-8. We associate this phenomenon to the physiological process of cell homeostasis^25,27^. This was also confirmed by the lack of immunoreactivity for cleaved Caspase-8 in the 13-week-old mice, both wild type and R6/2.

The expression of NLRP3 was significantly high in the 13-week-old R6/2 mouse striatum, where it was particularly distributed into the spiny projection neurons labeled by calbindin immunofluorescence. These are the neurons that degenerate massively in Huntington’s disease^11,28–30^.

The novelty of our data is represented by the finding that the degeneration of these neurons is sustained by pyroptosis, which can account, at least in part, for their degeneration. Our data were corroborated by our immunohistochemical and molecular study of the pyroptosis marker, namely, cleaved Caspase-1^31^, which confirmed that those cells were undergoing a pyroptotic cell death^32^.

Furthermore, we observed that striatal interneurons that are typically resistant to neurodegeneration in HD such as cholinergic neurons, expressed low levels of NLRP3, thus confirming that they were excluded from the ongoing inflammasome process and related pyroptosis-associated striatal neuronal degeneration. Conversely, evidence of the inflammasome complex, which is part of the pyroptosis pathway, was observed in the parvalbuminergic and calretininergic striatal neurons, which are prone to degenerate in HD^31,33^. Thus, we confirmed that healthy neurons did not show signs of pyroptosis, while degenerating ones did.

Clearly, further studies are necessary to investigate the role of caspase 1 in promoting cleavage of IL1β and IL-18.

We studied the distribution of inflammasome NLRP3 in microglia. An activation of microglia associated with neuroinflammation is observed in Huntington’s disease, and compounds that reduce microglia activation proved beneficial^12,13,22^. We observed here that reactive microglial cells were intensely stained for Iba1 in the 13-week-old R6/2 mice and devoid of NLRP3 immunoreactivity. NLRP3 immunoreactive product was observed in brain areas where microglia was activated, although we did not observe a colocalization. In a recent study^13^, microglial cells were found immunoreactive for NLRP3 in mice injected lentiviruses harboring shLgals3: Slight differences in the animal model used could explain this discrepancy. Moreover, it is conceivable that the inflammasome is contained in the degenerating cells rather than in those cells that facilitate this process.

However, our data support the evidence that reactive microglia is part of the inflammasome and contributes functionally to the pyroptosis process^34,35^.

Our interest in pyroptosis is driven by the concept that the inhibition of inflammatory response and pyroptosis was recently shown to counteract neurodegeneration in several disease models. Indeed, the inhibition of pyroptosis-mediated neurotoxicity is beneficial in cellular and animal AD models^36^. Moreover, cerebral ischemia-induced brain damage is alleviated by downregulating inflammatory response and pyroptosis mediated by Bromodomain-containing protein 4(BRD4)^10^.

With this in mind, we have demonstrated that pyroptosis plays a role in the degeneration of striatal neurons in HD, and we infer that counteracting the process and the inflammasome with appropriate compounds could be beneficial in fighting Huntington’s disease^37^.

## Materials and methods

### Animals and tissue processing

All animal experiments, which satisfied ARRIVE guidelines, were performed in accordance with European Communities Council Directive (2010/63 EU) as adopted by the Santa Lucia Foundation Animal Care and Use and approved by Italian Ministry of Health. Transgenic R6/2 mice were obtained by ovarian transplant of hemi zygote females × B6CBAF1/J males, all obtained from Jackson Laboratories (Bar Harbor, ME). F1 mice were used to perform all experiments. Ten mice per group were used. No randomization was performed. The study groups included: R6/2 mice at 4 weeks of age, R6/2 mice at 13 weeks of age, Wild-type mice at 4 and 13 weeks. Mice were handled under the same conditions by one investigator at the same day and time. Genotyping was performed at 21 days of age, and all mice were weaned and housed four in each cage under standard conditions (room temperature: 20 ± 2 °C; humidity: 60%) and a 12/12-h light/dark cycle (7:00 am–7:00 pm) with ad libitum access to food and water. Forty animals (10 R6/2 4 weeks, 10 R6/2 13 weeks, 10 wild type 4 weeks and 10 wild type 13 weeks) were transcardially perfused under deep anesthesia with saline solution containing 0.01 ml heparin, brains were removed and cut in half. One half brain was post-fixed in 14 ml of 4% paraformaldehyde, 10% sucrose and 20% glycerol in 0.1 M phosphate buffer (PB) at +4 °C for 48 h. Sectioning was performed on a sliding frozen microtome at 40 μm thickness. Observers who were in blind, collected primary data.

### Immunohistochemical studies

#### Evaluation of activated Caspase-8 and NLRP3

Coronal brain sections were incubated with the apoptotic marker Caspase-8 (polyclonal anti-Caspase-8, Abcam, Novus Biologicals, Italy). The antibody specifically recognized the cleaved and activated Caspase-8 protein. Antigen retrieval was performed in Citrate Buffer (pH 6) for 20 min at 80 °C. After that, sections were retained in this buffer solution while allowing it to cool at room temperature. Sections were rinsed three times for 5 min at room temperature and subsequently incubated with the primary antibody. The immunohistochemical staining was completed with the streptavidin-biotin amplification (Jackson Immunoresearch, West Grove, PA, USA). A set of sections was processed for the inflammasome marker NLRP3 (polyclonal anti-NLRP3, Abcam, Novus Biologicals, Italy) at 1:200 dilution in a 0.1 M PB solution containing 0.3% Triton X-100 for 72 h at 4 °C. All sections were rinsed three times for 5 min at room temperature and subsequently counterstained with Neurotrace^TM^ (Nissl-like fluorescent marker, Jackson, USA) to calculate the number and the immunofluorescence intensity of neurons containing Caspase-8 and NLRP3. Sections were mounted on specimen slides, cover slipped with GEL-MOUNT (Sigma-Aldrich, Italy) and a confocal laser scanning microscope (Zeiss LSM 800) was used to acquire all the images. A sample of about 100 neurons for each of three sections obtained by the 10 mice per group were analyzed, in order to determine the distribution of the cleavage product of Caspase-8 and the immunoreaction product of NLRP3 in the striatum of R6/2 and wild-type mice at the different time points 4 and 13 weeks.

#### Immunohistochemistry for Caspase-1

To evaluate the involvement of inflammasome complex in our system, we performed an immunohistochemistry for Caspase-1. Peroxidase-antiperoxidase diaminobenzidine tetrahydrochloride single-label immunohistochemistry for Caspase-1 was performed to identification and quantification of neurons undergoing pyroptosis in the striatum. Serial sections from rostral neostriatum through the level of anterior commissure (interaural 4.66 mm/Bregma 0.86 mm to interaural 3.34 mm/Bregma−0.46 mm) for three animals per groups, were incubated with mouse anti-Caspase-1 at 1:200 dilution in 0.1 M PB solution containing 0.3% Triton X-100 for 72 h at 4 °C. Subsequently, sections were incubated with mouse peroxidase–antiperoxidase complex diluted 1:100 in 0.1 M PB solution with 0.3% Triton X-100 at room temperature for 1 h. After peroxidase–antiperoxidase incubation, sections were incubated in Tris-Hcl buffer containing 10 mg diaminobenzidine tetrahydrochloride for 2 min, adding 15 µl of 3% hydrogen peroxidase. The peroxidase–antiperoxidase diaminobenzidine tetrahydrochloride-labeled sections were then washed in distillated water, placed in 0.1 M PB, mounted on gelatin-coated slides, dried, dehydrated and coverslipped. Caspase-1 positive cells count was performed on collected images obtained by Neurolucida^TM^ Stereo Investigator software (Zeiss, Rochester, NY, USA).

#### Western blotting

Dissected striata from the Wt and R6/2 half brains were homogenized with the RIPA lysis buffer containing a protease and phosphatase inhibitor cocktail (Sigma Aldrich, USA) and centrifuged at 13,000 × *g* for 20 min. Equal amounts of protein were separated using sodium dodecyl sulfate-polyacrylamide gel electrophoresis, transferred to polyvinylidene fluoride membranes, and incubated with Caspase-8 (polyclonal anti-Caspase-8, Abcam, Novus biologicals 1:1000), Caspase-1, which detects both procaspase 1 and its cleavage product (mouse anti-Caspase-1 (14F468) Novus biological 1:500) and mouse GAPDH (1:10,000; Sigma Aldrich, St Louis, MO) antibodies, overnight at 4 °C. After being washed with Tris-buffered saline (TBS)/Tween 20, membranes were incubated with HRP-labeled secondary antibody. Proteins signal was visualized using the Invitrogen iBright CL 1500 Imaging system.

#### Analysis of NLRP3 in striatal projection neurons

Double-label immunofluorescences were carried out to evaluate the distribution of NLRP3 in calbindin labeled striatal neurons and its expression levels in the mice striatum. Coronal brain sections of mice were incubated with a cocktail of anti-NLRP3 antibody and a mouse antibody against Calbindin protein (mouse anti-Calbindin, Abcam, Novus Biologicals, Italy) at 1:200 dilution in a 0.1 M PB solution containing 0.3% Triton X-100 for 72 h at 4 °C. After that, sections were rinsed three times for 5 min at room temperature and subsequently incubated with the Alexa Fluor 555 and 488 secondary antibodies (Immunological Science, Italy) for 2 h at room temperature at 1:200 dilution in a 0.1 M PB solution containing 0.3% Triton X-100. Sections were then mounted on slides, cover slipped with GEL-MOUNT (Sigma-Aldrich, Italy). A confocal laser scanning microscope (Zeiss LSM 800) was used to acquire all of the images. Three separate fields (dorsolateral, central and medial each 1 mm in diameter) in each of three rostro caudally spaced sections of ten mice per group were examined. NLRP3 and Calbindin immunofluorescence intensity was measured and quantified by using the Java image processing and analysis program available in, Fiji ImageJ. All confocal images were acquired under no saturation conditions, with a ×20 objective raising a ×1 zoom with value 0 of Offset and producing images in the format 1024 × 1024, Airy Units 1.0. The same set configuration was performed for all samples. Colocalization of NLRP3 in striatal projection neurons was calculated performing Coloc2 analysis, the plugin provided by Fiji ImageJ.

The mean Mander’s coefficients, obtained by the plugin Coloc2 performing Costes Autothresholds on samples regions of interest (ROIs) and running the analysis on 100 randomized images, varies from 0 to 1, corresponding to non-overlapping images and 100% or quite colocalization between the two images, respectively.

#### Striatal interneurons characterization

A double immunohistological staining for striatal interneurons markers and the pyroptosis marker NLRP3 was performed. Brain sections were incubated with goat anti-choline acetyl transferase (ChAT; Nova biological, CA, USA); mouse anti-calretinin (CALR; Chemicon International, Inc., Temecula, CA, USA); mouse anti-parvalbumin (PARV, Chemicon International, Inc., Temecula, CA, USA) and polyclonal anti-NLRP3. All primary antibodies were used at a 1:200 dilution, in 0.1 M PB containing 0.3% Triton X-100 for 72 h at 4 °C. Sections were rinsed three times for 5 min at room temperature and subsequently incubated with secondary antibodies Alexa Fluor 488 and 555 for 2 h at room temperature at 1:200 dilution in a 0.1 M PB solution containing 0.3% Triton X-100. Subsequently, sections were mounted on slides, cover slipped with GEL-MOUNT and examined under an epi-illumination fluorescence microscope (Zeiss Axioskop 2). The confocal laser scanner microscopy (Zeiss LSM800) was used to acquire images. Immunofluorescence intensity and colocalization analysis were performed by using the Java image processing and plugin analysis program included in Fiji ImageJ.

#### Microglial morphology

Microglial morphology was studied by a double immunostaining with an antibody for microglia (goat anti-Iba-1 from Novus Biologicals, Italy) and NLRP3. Striatal brain sections were incubated with the primary antibodies for 72 h at 4 °C, followed by incubation with the appropriate secondary antibodies for 2 h at room temperature. Images were acquired by confocal laser scanner microscopy (Zeiss LSM 800) in order to perform soma size analysis. Microglia cells in the area of interest were captured using a ×20 objective raising a ×1 zoom and images in the format 1024×1024, Airy Units 1.0 were produced. This configuration was used for all samples. Collected images were exported in TIFF format, brightness and contrast were adjusted. The area of soma of Iba-1 positive cells was characterized by using ×63 Z-stack images, performing the school analysis available in the Java image processing and analysis program Fiji Image J. The Iba-1 immunostained area was calculated ad Iba-1 area/total area analyzed and indicated as percentage.

#### Statistical analysis

All the collected images have been quantified by using the Java image processing and analysis program Fiji ImageJ. Cells of interest were selected using the freehand tool. From the Analyze menu, Set measurements Mean “Gray Value”, “Area” and “Min and Max Gray Value” were selected. The region characterized by absence of fluorescence was considered in the background and it was subtracted. Finally, the mean values with SEM were obtained for all measures. ANOVA analysis available in the software GraphPad Prism version 8.0 was performed. *P* values < 0.05 were considered statistically significant.
